# Role of lipoprotein-associated phospholipase A2 (LP-PLA2) in the prediction and assessment of the severity of coronary artery disease in patients with type 2 diabetes mellitus

**DOI:** 10.5937/jomb0-57486

**Published:** 2025-10-28

**Authors:** Irena Kostovska, Svetlana Cekovska, Katerina Tosheska'Trajkovska, Danica Labudovic, Julijana Brezovska-Kavrakova, Sonja Topuzovska, Hristina Ampova, Melda Emin, Elena Petrushevska-Stanojevska, Natasa Nedeska-Minova, Ognen Kostovski, Marjan Boshev

**Affiliations:** 1 Institute of Medical and Experimental Biochemistry, Faculty of Medicine, Ss. Cyril and Methodius University in Skopje, Republic of North Macedonia; 2 City General Hospital "8th September" - Department of Endocrinology, Diabetes and Metabolic Diseases, Skopje, Republic of North Macedonia; 3 University Clinic of Digestive Surgery, Faculty of Medicine, Ss Cyril and Methodius University of Skopje, North Macedonia; 4 University Clinic of Cardiology, Faculty of Medicine, Ss Cyril and Methodius University of Skopje, North Macedonia

**Keywords:** lipoprotein-associated phospholipase A2, coronary artery disease, type 2 diabetes mellitus, fosfolipaza A2 povezana s lipoproteinima, koronarna arterijska bolest, dijabetes melitus tipa 2

## Abstract

**Background:**

Lipoprotein-associated phospholipase A2 (Lp-PLA2) is a specific biomarker associated with an increased risk of coronary artery disease (CAD) development. This study aims to determine the relationship between Lp-PLA2 activity and the risk of development and severity of CAD in patients with type 2 Diabetes mellitus (T2DM).

**Methods:**

The cross-sectional study included 148 patients with T2DM, divided into two groups: patients with T2DM without confirmed CAD (n=56) and patients with T2DM and confirmed CAD (n=92), further divided into three sub-groups based on the stage of CAD, and a control group of healthy individuals (n=44). Venous blood samples were collected from all participants to measure glucose, cholesterol, triglycerides, HDL, LDL, C-reactive protein, urea, and creatinine levels using standard photometric methods. Lp-PLA2 activity was measured using a chemiluminescent immunoassay method.

**Results:**

Patients with T2DM and confirmed CAD had significantly higher Lp-PLA2 levels than those without confirmed CAD and healthy individuals. A significant difference in Lp-PLA2 levels was found between the group without CAD, the patients with CAD divided into subgroups according to disease stage, and the healthy control group. A positive correlation was observed between Lp-PLA2 and BMI, glycated haemoglobin, total cholesterol, and HDL cholesterol. The optimal cutoff value for Lp-PLA2&lt;250 ng/mL yielded a diagnostic sensitivity of 95.65% and specificity of 88.64% for patients with T2DM and diagnosed CAD.

**Conclusions:**

Lp-PLA2 can be used as a predictor for developing and assessing the severity of CAD in patients with T2DM.

## Abstract (sr)

**Uvod:**

Fosfolipaza A2 povezana s lipoproteinima (Lp-PLA2) predstavlja specifičan biomarker povezan s povećanim rizikom od razvoja koronarne arterijske bolesti (CAD). Cilj ove studije bio je da se utvrdi veza između aktivnosti Lp-PLA2 i rizika od razvoja i težine CAD kod pacijenata sa dijabetesom tipa 2 (T2DM).

**Metode:**

U studiji preseka je učestvovalo 148 pacijenata sa T2DM podeljenih u dve grupe: pacijenti sa T2DM bez potvrđene CAD (n=56) i pacijenti sa T2DM i potvrđenom CAD (n=92), koji su dalje podeljeni u tri podgrupe na osnovu stadijuma bolesti, kao i kontrolna grupa zdravih osoba (n=44). Od svih učesnika su uzeti venski uzorci krvi radi određivanja nivoa glukoze, holesterola, triglicerida, HDL-a, LDL-a, C-reaktivnog proteina, uree i kreatinina pomoću standardnih fotometrijskih metoda. Aktivnost Lp-PLA2 određena je metodom hemiluminiscentnog imunoeseja.

**Rezultati:**

Pacijenti sa T2DM i potvrđenom CAD su imali značajno više vrednosti Lp-PLA2 u poređenju sa pacijentima bez potvrđene CAD i zdravim osobama. Uočena je značajna razlika u nivou Lp-PLA2 između grupe bez CAD, pacijenata sa CAD raspoređenih po stadijumima bolesti i kontrolne grupe. Primećena je pozitivna korelacija između Lp-PLA2 i indeksa telesne mase, glikoziliranog hemoglobina, ukupnog holesterola i HDL holesterola. Optimalna granična vrednost Lp-PLA2&lt;250 ng/mL pokazala je dijagnostičku osetljivost od 95,65% i specifičnost od 88,64% za pacijente sa T2DM i dijagnostikovanom CAD.

**Zaključak:**

Lp-PLA2 se može koristiti kao prediktor za razvoj i procenu težine CAD kod pacijenata sa T2DM.

## Introduction

Type 2 diabetes mellitus has reached global epidemic proportions. Coronary artery disease (CAD) is the most common complication and the leading cause of mortality in patients with type 2 diabetes mellitus [1]. According to the World Health Organization, the mortality rate from CAD among patients with type 2 diabetes mellitus is 50% [2]. The Framingham risk score includes well-established risk factors for CAD, such as age, sex, serum cholesterol, LDL, HDL, and cigarette smoking. Diabetes mellitus is also recognised as a contributing factor. Blood biomarkers serve as valuable laboratory tools for assessing the risk and severity of CAD. Several potential biomarkers for predicting CAD in type 2 diabetes mellitus patients have been identified, including high-sensitivity C-reactive protein (hs-CRP), interleukin-6 (IL-6), monocyte chemoattractant protein-1 (MCP-1), tumour necrosis factor-alpha (TNF-α), vascular endothelial growth factor (VEGF), reactive oxygen species (ROS), protein kinase C, and nuclear factor-kB. However, recent scientific attention has increasingly focused on lipoprotein-associated phospholipase A2 (Lp-PLA2) [3].

Lp-PLA2 is an enzyme with a molecular mass of 45 kDa, serving as a vascular inflammatory marker with a significant role in atherogenesis and the development of CAD. Recent epidemiological studies suggest that elevated Lp-PLA2 levels are closely associated with an increased risk of CAD [4]. Lp-PLA2 is primarily secreted by macrophages, lymphocytes, and foam cells within atherosclerotic plaques. It binds to LDL and remains inactive until LDL oxidation occurs. Once oxidised, LDL contains oxidised phosphatidylcholine, a substrate for Lp-PLA2. This reaction produces two highly inflammatory mediators: lysophosphatidylcholine and oxidised non-esterified free fatty acids. In the bloodstream, approximately 70% of Lp-PLA2 is bound to apolipoprotein B (apoB) from LDL and lipoprotein(a) (Lp(a)), while 30% is bound to apolipoprotein A (apoA) from HDL. Patients with diabetes mellitus typically exhibit elevated LDL levels [5]. Lp-PLA2 contributes to atherogenesis primarily by increasing endothelial infiltration of coronary arteries by macrophages, the primary source of this enzyme. Additionally, oxidised phospholipids promote inflammation and atherogenesis, leading to endothelial dysfunction, plaque formation, plaque necrosis, and heightened oxidative stress in T2DM patients due to hyperglycemia. Lp-PLA2 is correlated with oxidative stress and progenitor cell dysfunction, microvascular dysfunction, and impaired reverse cholesterol transport in type 2 diabetes mellitus patients [1] [5] [6] [7] [8].

Numerous epidemiological studies indicate that Lp-PLA2 is a predictive biomarker for CAD. According to some studies, Lp-PLA2 is a more effective marker for early detection of CAD risk than high-sensitivity CRP [9]. In patients with low coronary risk (classified according to the Framingham risk score), Lp-PLA2 has been identified as a significant independent predictor of CAD in type 2 diabetes mellitus patients. It is closely associated with CAD severity [5]. This suggests that Lp-PLA2 may be crucial for detecting subclinical CAD.

Additionally, studies have found a positive correlation between Lp-PLA2 levels and the number of atherosclerotic plaques in the coronary arteries of T2DM patients [6]. Some studies have found a positive correlation between Lp-PLA2 and fasting blood glucose levels in type 2 diabetes mellitus patients. The same study observed a positive correlation between oxLDL and plasma glucose concentration.

Furthermore, insulin resistance and Lp-PLA2 activity have established a positive correlation. Serum Lp-PLA2 activity is positively associated with lysophosphatidylcholine (LPC) concentration in LDL among type 2 diabetes mellitus patients [10] [11] [12] [13]. According to the latest WHO data, the number of people with diabetes reached 830 million in 2022. The prevalence has risen more rapidly in low- and middle-income countries than in high-income countries. Diabetes treatment coverage is lowest in low- and middle-income countries. Around 11% of cardiovascular deaths were caused by high blood glucose levels [14].

The treatment of both type 2 diabetes mellitus and CAD, the most common complications among diabetes patients, and the disability associated with these conditions have a significant economic impact on society. Introducing new biomarkers like Lp-PLA2 into routine laboratory practice is crucial for predicting CAD in T2DM patients. Early detection, especially in the subclinical stage, can help delay or prevent CAD onset and ensure timely treatment.

Thus, the main objective of this study is to determine the relationship between Lp-PLA2 activity and the risk of development and severity of CAD in patients with T2DM.

## Materials and methods

### Study design and participants

The study design is a cross-sectional study, including 148 patients with a confirmed diagnosis of type 2 diabetes mellitus (T2DM), with and without coronary artery disease (CAD), and a control group of healthy individuals (n=44, 50% male and 50% female). The study participants were between 35 and 65 years old. The study was conducted from 2022 to 2025. Patients with T2DM were divided into two groups: those without confirmed CAD (n=56, 65% male, 35% female) and those with confirmed CAD by coronary angiography (n=92, 61% male, 39% female). Patients with T2DM and confirmed CAD were further subdivided into three groups based on the stage of CAD:

- First subgroup (n=26, 68% male, 32% female): Patients with chest pain and fatigue symptoms but with normal coronary angiography findings.
- Second subgroup (n=37, 57% male, 43% female): Patients with chest pain and fatigue symptoms, with intermediate lesions up to 70% stenosis of the vessel lumen as determined by coronary angiography.
- Third subgroup (n=29, 59% male, 41% female): Patients with symptoms of chest pain and fatigue, presenting with acute coronary syndrome (STEMI – ST-segment elevation myocardial infarction, or NON-STEMI) and coronary angiography findings of 95% stenosis up to and/or 100% occlusion of the coronary artery lumen.

Patients with T2DM without confirmed CAD and some with confirmed CAD were selected from the Department of Endocrinology, Diabetes, and Metabolic Disorders at the City General Hospital on 8th September – Skopje. Patients with T2DM and confirmed coronary heart disease were selected from the University Clinic of Cardiology. Blood samples from healthy individuals were obtained from volunteers participating in clinical studies at the Institute of Pharmacology and Toxicology, Medical Faculty, Skopje.

Inclusion criteria for patients with T2DM with and without confirmed CAD were the duration of T2DM of at least 10 years and the confirmed presence or absence of CAD through coronary angiography. Exclusion criteria included the presence of other cardiovascular diseases.

During the examination, data on age, gender, body weight, height, blood pressure, glycemic control, duration of diabetes, severity of coronary heart disease and interventions, comorbidities, alcohol consumption, smoking, and medication use were recorded in a questionnaire. Body mass index (BMI) was calculated by dividing body weight (in kg) by height (in cm) and expressed as square meters.

### Ethics

All participants provided written informed consent before inclusion in the study. The study was conducted by the ethical principles of the Declaration of Helsinki and was approved by the Ethics Committee of the Medical Faculty in Skopje, Republic of North Macedonia (Approval No. 03-5602/11 from 16.12.2022).

### Measurement of biochemical parameters and activity of Lp-PlA2

Venous blood samples were collected from all participants in vacutainer tubes without anticoagulant for serum separation. Serum samples were analysed in the Laboratory for Biochemical Investigations at the Institute of Medical and Experimental Biochemistry, Medical Faculty, Skopje, and the Biochemical Laboratory at the City General Hospital 8th September - Skopje. Standard photometric methods using an automated analyser Cobas 600 (Roche, Basel, Switzer land) were applied to measure glucose, cholesterol, triglycerides, HDL, LDL cholesterol, C-reactive protein, urea, and creatinine concentrations. Lp-PLA2 concentration was measured at the Laboratory for Biochemical Investigations at the Institute of Medical and Experimental Biochemistry, Medical Faculty, Skopje, using a chemiluminescence immunoassay (CLIA) method with the fully automated Snibe Maglumi 800 analyser (Snibe Diagnostic, Shenzhen New Industries Biomedical Engineering Co. Ltd., Shenzhen, China).

### Statistical analysis of data

The collected data were statistically analysed using MedCalc for Windows, Version 23.1.7 (MedCalc Software, Ostend, Belgium). Categorical data were presented as numbers and percentages, while quantitative data were expressed as arithmetic mean and standard deviation. The following statistical tests were used: Fisher’s Exact Test, Kolmogorov-Smirnov test, One-way analysis of variance (ANOVA), Kruskal-Wallis test, and Pearson correlation coefficient. Receiver Operating Characteristic (ROC) analysis was used to determine the diagnostic sensitivity and specificity of Lp-PLA2 in patients with T2DM and CAD. A p-value<0.05 was considered statistically significant.

## Results

### Comparison of clinical and laboratory data among study groups of T2DM patients divided into patients with and without proven CAD and healthy subjects

The study results revealed significant differences among groups, including patients with T2DM (with and without CAD) and healthy individuals, regarding the following clinical and laboratory parameters: age, blood glucose concentration, glycated haemoglobin (HbA1c), systolic blood pressure (SBP), total cholesterol, triglycerides, HDL, LDL cholesterol, urea, serum creatinine, CRP, and Lp-PLA2. However, no statistically significant difference was found among the groups for the following parameters: body mass index (BMI), disease duration, and diastolic blood pressure (DBP). The comparison results of the clinical and laboratory data among patients with T2DM categorised into those with and without CAD and healthy individuals, are presented in Table 1.

**Table 1:** Comparison of the clinical and laboratory data among patients with T2DM, divided into those with and without CAD and control group. Results are presented as mean ± standard deviation (SD).<br>Abbreviations: T2DM, type 2 diabetes mellitus; HbA1c, glycated haemoglobin; BMI, body mass index; Lp-PLA2, lipoprotein-associated phospholipase A2; SBP, systolic blood pressure; DBP, diastolic blood pressure; HDL, high-density lipoproteins; LDL, low-density lipoproteins; CRP, C-reactive protein.

Clinical and<br>laboratory data	Patients with T2DM<br> without CAD<br>n=56	Patients with T2DM<br>with CAD<br>n=92	Control group<br>n=44	p-value
Age (years)	58.2±6.005	54.6±6.4	40.5±8.13	<0.001
BMI (kg/m^2^)	28.93±0.67	27.8±0.74	26.5±0.68	0.029
Duration of T2DM (years)	6.78±2.62	7.1±2.75	/	0.324
Smoking	68%	62%	54%	0.367
Alcohol	41%	37%	28%	0.325
Blood glucose (mmol/L)	7.46±1.6	7.61±1.45	4.73±0.8	<0.001
HbA1c (%)	7.45±1.45	6.46±1.5	4.36±0.5	<0.001
SBP (mm/Hg)	155.5±2.23	157.04±2.14	125±1.54	<0.001
DBP (mm/Hg)	87.7±0.95	91.93±1.06	87.7±0.95	0.0179
CRP (mg/dL)	111.86±9.08	135.25±10.05	2.25±0.13	<0.001
Total cholesterol (mmol/L)	6.93±1.38	7.14±1.36	4.93±0.08	<0.001
Triacylglycerols (mmol/L)	5.02±2.2	5.08±3.02	4.08±1.23	<0.001
HDL (mmol/L)	1.32±0.76	1.49±0.76	5.9±1.08	<0.001
LDL (mmol/L)	4.7±1.54	4.54±2.14	3.3±0.08	<0.001
Blood urea (mmol/L)	7.83±2.78	8.4±3.1	4.4±2.1	<0.001
Serum creatinine (μmol/L)	128.5±5.2	135.5±3.07	66.7±2.08	<0.001
Lp PLA2 (ng/mL)	280.95±8.65	820.08±31.76	147.64±7.08	<0.001

Further statistical analysis showed significant differences among subgroups of T2DM patients without CAD and those with CAD (classified into three subgroups based on the stage of CAD), as well as the healthy individuals, concerning the following clinical and laboratory parameters: age, blood glucose concentration, HbA1c, systolic and diastolic blood pressure (SBP, DBP), total cholesterol, triglycerides, LDL cholesterol, CRP, and Lp-PLA2. No statistically significant difference was found among the subgroups regarding alcohol consumption, BMI, HDL cholesterol, disease duration, blood urea, and serum creatinine. The comparison results among the subgroups of T2DM patients without CAD, those with CAD (classified into three subgroups based on CAD stage), and healthy individuals are presented in Table 2.

**Table 2:** Comparison of clinical and laboratory data among patients with T2DM categorised into those without and with CAD, is subdivided into the CAD and control group stages. Results are presented as mean ± standard deviation (SD).<br>Abbreviations: T2DM, type 2 diabetes mellitus; HbA1c, glycated haemoglobin; BMI, body mass index; Lp-PLA2, lipoprotein-associated phospholipase A2; SBP, systolic blood pressure; DBP, diastolic blood pressure; HDL, high-density lipoproteins; LDL, low-density lipoproteins; CRP, C-reactive protein.

Clinical and<br>laboratory data	Patients<br>with T2DM<br>without CAD<br>n=56	Patients with<br>T2DM with<br>CAD in Stage 3<br>n=29	Patients with<br>T2DM with<br>CAD in Stage 2<br>n=37	Patients with<br>T2DM with<br>CAD in Stage 1<br>n=26	Control group<br>n=44	p-value
Age (years)	58.2±6.005	55.27±6.67	55.27±6.42	52.8±5.7	40.5±8.13	<0.001
BMI (kg/m^2^)	28.93±0.67	28.09±5.07	29.9±4.33	28.36±2.12	26.5±0.68	0.0321
Duration of T2DM (years)	6.78±2.62	7.27±2.34	7.62±3.06	6.19±2.59	/	0.177
Smoking	68%	58%	54%	60%	54%	<0.001
Alcohol	41%	52%	38%	41%	28%	0.042
Blood glucose (mmol/L)	7.46±1.6	7.49±2.5	7.7±2.8	6.8±2.6	4.73±0.8	<0.001
HbA1c (%)	7.45±1.45	8.06±1.46	7.89±1.94	6.19±1.26	4.36±0.5	<0.001
SBP (mm/Hg)	155.5±2.23	156.37±1.02	160.27±2.05	150.6±1.22	125±1.54	<0.001
DBP (mm/Hg)	87.7±0.95	92.41±0.98	92.14±1.06	92.85±1.24	87.7±0.95	<0.001
CRP (mg/dL)	13.11±6.6	11.03±5.65	12.2±6.31	11.17±6.69	2.25±0.13	<0.001
Total cholesterol (mmol/L)	6.93±1.38	7.26±2.31	5.55±2.25	5.21±2.72	4.93±0.08	<0.001
Triacylglycerols (mmol/L)	5.02±2.2	4.37±1.91	4.48±2.65	4.07±1.93	4.08±1.23	<0.001
HDL (mmol/L)	1.32±0.76	1.43±1.5	1.64±1.6	1.5±1.504	5.9±1.08	0.023
LDL (mmol/L)	4.7±1.54	4.03±2.14	3.66±2.33	3.8±1.95	3.3±0.08	<0.001
Blood urea (mmol/L)	7.83±2.78	6.75±2.23	6.27±2.22	6.55±1.96	4.4±2.1	0.008
Serum creatinine (μmol/L)	128.5±5.2	126.55±6.7	121.21±12.1	109.11±9.5	66.7±2.08	0.065
Lp PLA2 (ng/mL)	280.95±8.65	867.6±22.5	552.89±21.1	230.02±10.2	147.64±7.08	<0.001

### Correlation between Lp-PLA2 activity and clinical and laboratory parameters in studied subjects

Spearman’s correlation test was conducted to determine the correlation coefficient between Lp-PLA2 levels and laboratory parameters. A significant positive correlation was found between Lp-PLA2 and BMI (r=-0.347, p << 0.001) and between Lp-PLA2 and glycated haemoglobin (r=0.347, p<0.001). Lp-PLA2 activity also positively correlated with total cholesterol (r=0.14, p=0.07) and HDL cholesterol (r=0.15, p=0.06). No correlation was found between Lp-PLA2 and the following parameters: age, blood glucose concentration, triglycerides, LDL cholesterol, urea, serum creatinine, systolic and diastolic blood pressure, CRP concentration, and disease duration. The correlation coefficients between Lp-PLA2 activity and clinical and laboratory data in patients are presented in Table 3.

**Table 3:** Correlation between Lp-PLA2 levels and clinical and laboratory data of subjects. Abbreviations: T2DM, type 2 diabetes mellitus; HbA1c, glycated haemoglobin; BMI, body mass index; Lp-PLA2, lipoprotein-associated phospholipase A2; SBP, systolic blood pressure; DBP, diastolic blood pressure, HDL, high-density lipoproteins; LDL, low-density lipoproteins; CRP, C-reactive protein.

Clinical and<br>laboratory data	Lp-PLA2	
	r	p
Age (years)	-0.077	0.34
Duration of disease (years)	0.095	0.248
BMI (kg/m^2^)	0.347	<0.001
Glucose (mmol/L)	0.007966	0.9234
HbA1c (%)	0.347	<0.001
Total cholesterol (mmol/L)	0.1455	0.0777
Triglycerides (mmol/L)	0.05041	0.5429
HDL (mmol/L)	0.1523	0.0647
LDL (mmol/L)	-0.02741	0.7409
Blood urea (mmol/L)	-0.08254	0.3186
Serum creatinine (μmol/L)	-0.08040	0.3314
SBP (mm/Hg)	0.01391	0.8667
DBP (mm/Hg)	-0.0546	0.5102
CRP (mg/dL)	-0.06984	0.399

### Determination of diagnostic sensitivity and specificity of Lp-PLA2 in patients with T2DM and CAD

**Figure 1:**
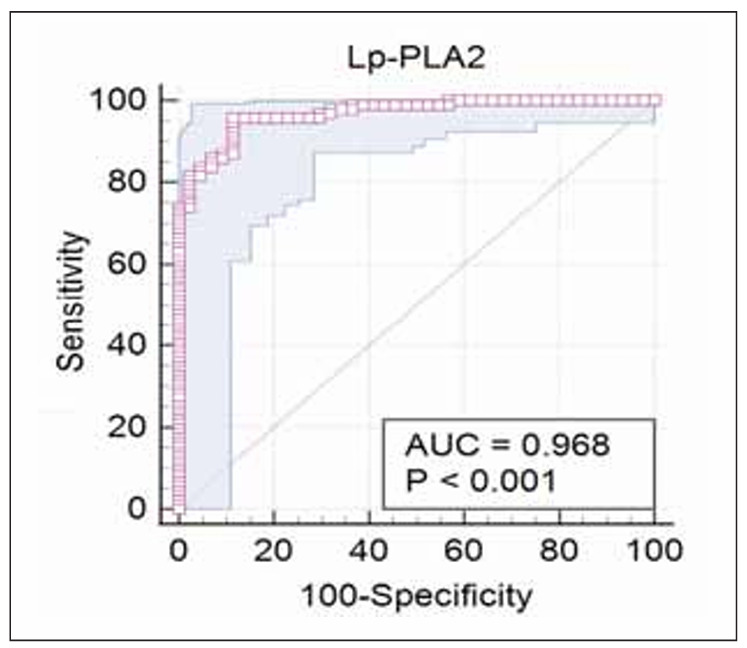
Diagnostic sensitivity and specificity of Lp-PLA2 in patients with T2DM and CAD.

A non-parametric ROC analysis was performed to assess the diagnostic value of Lp-PLA2 in patients with T2DM. The optimal cutoff value for Lp-PLA2<250 ng/mL (95th percentile) yielded a diagnostic sensitivity of 95.65%, specificity of 88.64%, negative predictive value of 99.4%, and positive predictive value of 48.3%. Table 4 and Figure 1 present the diagnostic sensitivity and specificity of Lp-PLA2 in patients with T2DM and CAD.

**Table 4:** Diagnostic sensitivity and specificity of Lp-PLA2 in patients with T2DM and CAD. Abbreviations: NPV, negative predictive value; PPV, positive predictive value.

The area under the ROC curve (AUC)	0.968
95% Confidence interval (95% CI)	0.923–0.991
Significance level p (Area=0.5)	<0.0001
Youden index J	0.8429
Optimal cut-off	<250 ng/mL
Sensitivity %	95.65
Specificity %	88.64
NPV - negative predictive value %	99.4
PPV – positive predictive value %	48.3

### Elevated Lp-PLA2 levels in T2DM patients with and without CAD, divided into CAD stages, and healthy individuals

**Figure 2:**
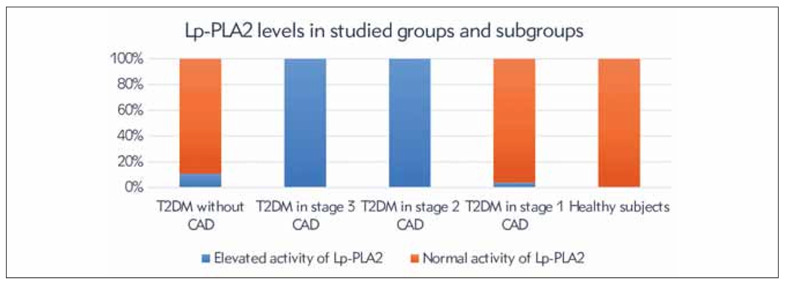
Elevated Lp-PLA2 levels in T2DM patients with and without CAD, categorised by CAD stages, and healthy subjects.

Lp-PLA2 levels were above the cutoff value in all patients with T2DM and CAD in stages 3 and 2, in 3.8% of patients in stage 1 of CAD, and in 10.7% of patients with T2DM without CAD. All healthy individuals had normal Lp-PLA2 activity. These results are illustrated in Figure 2.

## Discussion

Type 2 diabetes mellitus (T2DM) is a chronic metabolic condition that leads to microvascular and macrovascular complications, causing significant morbidity and mortality, which creates a substantial economic burden for countries worldwide. Patients with T2DM have a two to four times increased risk of developing cardiovascular diseases (CVD). In patients with low coronary risk (classified according to the Framingham risk score), Lp-PLA2 has been identified as a significant independent predictor of coronary artery disease (CAD) in patients with type 2 diabetes. It is closely associated with the severity of CAD [5].

The mechanisms of cardiovascular disease in T2DM resemble those in the general population but have unique characteristics. Inflammation is key in the pathogenesis of T2DM and CVD, highlighting a biological link between these conditions. Various circulating inflammatory markers have been extensively studied for their role as risk predictors for the development of CVD. Among these markers, lipoprotein-associated phospholipase A2 (Lp-PLA2) has attracted significant interest in recent decades. The endothelial cell layer acts as a barrier that separates circulating components from the arterial intima. It is believed that macrophage infiltration is increased in the arterial walls of patients with T2DM. Macrophages primarily secrete lp-PLA2. These macrophages infiltrate adipose tissue, produce oxidative byproducts, and increase Lp-PLA2 production. Free radicals generated during glucose oxidation can enhance lipoprotein oxidation, thereby increasing the substrate for Lp-PLA2. Intensive hypoglycemic therapy can reduce the overall activity of Lp-PLA2 and redistribute Lp-PLA2 activity towards a higher proportion in high-density lipoproteins (HDL). This phenomenon suggests that Lp-PLA2 activity is increased in hyperglycemic individuals at high risk for CAD.

Clinical studies show that patients with T2DM and metabolic disorders have higher trim, dense LDL levels, potentially providing a better matrix for Lp-PLA2 transport in circulation [7]. Many prospective studies also indicate that Lp-PLA2 is an independent predictor of coronary artery disease (CAD) [15].

Our study found that patients with T2DM and confirmed CAD had significantly higher Lp-PLA2 values, particularly those in the second and third stages of CAD, compared to patients with type 2 diabetes without confirmed CAD and healthy individuals. Similar data have been published in the study by Zhang N and colleagues [6] [16]. We found a statistically significant positive correlation between Lp-PLA2 and BMI (r=-0.347, p<0.001) and between Lp-PLA2 and HbA1c (r=0.347, p=<0.001), indicating that a high body mass index and poorly controlled T2DM further contribute to the increase in Lp-PLA2 activity [16]. A positive correlation was also established between Lp-PLA2 activity and HDL cholesterol, which is similar to the study’s findings by Sai Giridhar Sairam and collaborators. The diagnostic sensitivity and specificity values of Lp-PLA2 in patients with CAD are very similar to the specificity and sensitivity values found in the same study [17]. All patients in the third and second stages of CAD had elevated Lp-PLA2 values, while all healthy subjects had normal Lp-PLA2 levels. In patients with T2DM without diagnosed CAD, we found elevated values in 10.7%, suggesting that regular cardiology check-ups in T2DM patients are necessary, as these cases likely represent a subclinical form of CAD. This indicates that Lp-PLA2 could be a valuable biomarker for the early detection of subclinical CAD. The low percentage of patients diagnosed with CAD in stage 1 in our study is likely due to the small number of participants in this group. This is one of the study’s limitations, as there was an unequal distribution of participants with T2DM and diagnosed CAD across different stages and a small sample size in all analysed groups and subgroups. Another limitation of the study is its design, which prevents follow-up of participants, particularly those without diagnosed CAD but with elevated Lp-PLA2 values. Therefore, in the future, a more extensive prospective study is needed, including a large number of participants equally represented across all CAD stages, with monitoring of Lp-PLA2 concentrations to obtain a clearer picture, particularly regarding the critical Lp-PLA2 value in patients in the early stages of CAD and those with T2DM who have no CAD symptoms and/or have not undergone coronary angiography.

## Conclusions

Lp-PLA2 can serve as a predictor for the occurrence and assessment of CAD severity in patients with T2DM. This would enable the early detection of T2DM patients with a subclinical form of the disease who are at high risk for developing CAD. This would enable timely therapeutic interventions to delay or prevent CAD onset.

The main conclusions are derived from the statistical analysis of the obtained data from the study:

- A significant difference in Lp-PLA2 activity was observed between T2DM patients (with and without CAD) and healthy controls.
- A significant difference between subgroups of patients with T2DM without CAD and those with CAD, divided into three subgroups according to CAD stages and healthy individuals in terms of Lp-PLA2 activity.
- A significant positive correlation between Lp-PLA2 and BMI and between Lp-PLA2 and HbA1c indicates that a high body mass index and poorly controlled T2DM further contribute to increased Lp-PLA2 activity.
- High diagnostic sensitivity and specificity of Lp-PLA2 in patients with type 2 diabetes and CAD.
- All patients with T2DM in the second and third stages of CAD had elevated Lp-PLA2 values, whereas all healthy participants in the study had normal Lp-PLA2 values.
- A high percentage of T2DM patients with elevated Lp-PLA2 values but without diagnosed (subclinical) CAD.

## Dodatak

### Conflict of interest statement

All the authors declare that they have no conflict of interest in this work.
